# Association between antibiotics use and osteoporotic fracture risk: a nationally representative retrospective cohort study

**DOI:** 10.1007/s11657-024-01438-8

**Published:** 2024-08-30

**Authors:** Ji Won Lee, Sun Jae Park, Young Jun Park, Seogsong Jeong, Jihun Song, Hye Jun Kim, Jooyoung Chang, Kyae Hyung Kim, Ji Soo Kim, Yun Hwan Oh, Yoosun Cho, Sang Min Park

**Affiliations:** 1 Department of Biomedical Sciences, Seoul National University Hospital, Seoul National University College of Medicine, Seoul, South Korea; 2 Department of Health Convergence, Ewha Womans University, Seoul, South Korea; 3 Medical Research Center, Genomic Medicine Institute, Seoul National University, Seoul, South Korea; 4 Department of Biomedical Informatics, Korea University College of Medicine, Seoul, South Korea; 5 Department of Family Medicine, Seoul National University Hospital, Seoul National University College of Medicine, Seoul, South Korea; 6 Public Healthcare Center, Seoul National University Hospital, Seoul, South Korea; 7 International Healthcare Center, Seoul National University Bundang Hospital, Seongnam, South Korea; 8 Department of Family Medicine, Chung-Ang University Gwangmyeong Hospital, Chung-Ang University College of Medicine, Gwangmyeong-Si, South Korea; 9 Total Healthcare Center, Kangbuk Samsung Hospital, Sungkyunkwan University School of Medicine, Seoul, South Korea

**Keywords:** Antibiotics, Osteoporotic fracture, Cohort, Epidemiology, Pharmacoepidemiology

## Abstract

***Summary*:**

This population-based retrospective cohort study aimed to estimate the association between antibiotic exposure and osteoporotic fracture risk. Long-term antibiotic use was associated with the risk of osteoporotic fracture. An increase in the number of antibiotic classes prescribed may also be associated with an increased osteoporotic fracture risk.

**Purpose:**

This study aims to examine the association between antibiotic usage and osteoporotic fractures in a large cohort of Korean adults, with a specific focus on the duration of antibiotic exposure and the number of antibiotic classes used.

**Methods:**

This retrospective cohort study from the National Health Insurance Service-National Health Screening Cohort (NHIS-HEALS) database from January 1, 2002, to December 31, 2019, included 167,370 Korean adults aged 50 years or older (mean [SD] age, 59.3 [7.82] years; 65,425 [39.09%] women). The cumulative antibiotic prescription days and the classes of antibiotics prescribed between 2004 and 2008 were exposure variables, respectively. The main outcome was a newly diagnosed osteoporotic fracture during follow-up. Cox proportional hazard regression was used to determine the adjusted hazard ratios (aHRs) and 95% confidence intervals (CIs) for the incident osteoporotic fractures associated with antibiotic exposure.

**Results:**

The antibiotic user group with 91 days had a higher risk of osteoporotic fracture in comparison to the antibiotic non-user group (aHR, 1.12; 95% CI, 1.03–1.21). Additionally, those who used more than four different antibiotic classes had an elevated risk of osteoporotic fracture compared to the non-user group (aHR, 1.10; 95% CI, 1.02–1.18).

**Conclusion:**

This extensive population-based cohort study conducted on a large population has identified an association between the utilization of antibiotics and an elevated risk of osteoporotic fractures. The cumulative days exposed to antibiotics and osteoporotic fractures may be positively associated.

**Supplementary Information:**

The online version contains supplementary material available at 10.1007/s11657-024-01438-8.

## Introduction

Osteoporosis is a medical condition that is distinguished by reduced bone density, degradation of bone structure, and disturbance of bone microarchitecture. This can lead to weakened bone strength and a heightened susceptibility to fractures [1]. As a result of the growing life expectancy, it is expected that the public health burden of osteoporotic fractures will grow in the forthcoming generations [2]. It is expected that by the year 2025, the worldwide incidence of osteoporotic fractures will rise to 3 million [3]. Between 2008 and 2016, there was a gradual increase in the incidence of osteoporotic fractures in the hip, humerus, vertebra, and distal radius among individuals aged 50 years and older in the Korean population, irrespective of gender [4]. Hence, it is imperative to recognize and mitigate the risk factors related to osteoporotic fractures to soothe the prevalence of osteoporotic fractures.

Fractures frequently result from physical activity, including repetitive motion or falls from elevated surfaces. However, an unstable metabolism may also be a contributing factor. According to recent studies, there is an association between gut microbiota and osteoporotic fracture [5, 6]. The microbiota has the potential to cause numerous diseases that can impact both local and distant organ systems [7]. The maintenance of homeostasis at the intestinal mucosa and beyond is dependent on the configuration of the gut microbial networking and the existence or non-existence of significant species that can trigger specific responses [8, 9]. Therefore, it is essential to consider the overall balance of the gut microbiota [10].

Furthermore, research has demonstrated that the utilization of antibiotics results in a dysbiosis of the gut microbiota [11–13]. According to a review study, there is evidence to suggest that antibiotic exposure among children is linked with a decrease in microbiota richness and diversity, as well as a shift in species balance [14]. This shift results in a decrease in the population of commensal bacteria that are deemed advantageous [14]. The review primarily encompasses research that concentrates on microbiota modifications that transpired within a brief duration of antibiotic prescription, specifically less than 1 month [14]. A study exploring relationships between gut microbiota composition, bone metabolism, and risk of fracture was conducted in Japan involving 38 postmenopausal women [15]. The study found that specific microbiota may have a role in bone metabolism and the risk of fracture [15]. Further studies assessing the association between antibiotic exposure and fracture risk in large populations are needed to verify these results.

This population-based retrospective cohort study utilized the Korean National Health Insurance Service-Health Screening Cohort (NHIS-HEALS) database to investigate the association between antibiotic exposure and the probability of developing osteoporotic fractures. The cohort study included Korean adults aged 50 years or older with 11 years of follow-up.

## Materials and methods

### Study population

From January 1, 2002, to December 31, 2019, a study was conducted on a cohort of individuals utilizing the NHIS-HEALS database of South Korea (NHIS-2022–2-341), consisting of demographic information, health screening data, clinical data, diagnoses, prescriptions, hospital visits, admissions, and mortality data. The NHIS database is widely utilized for epidemiological investigations on population health, and its precision has been recognized in other scholarly inquiries [16–18].

We included 272,416 individuals aged 50 or older who underwent health examinations between 2007 and 2008. We excluded individuals who had died (*n* = 1,077), who had previously experienced osteoporotic fractures (*n* = 13,254), who had previously taken bisphosphonate medication (*n* = 51,436), who had previously been diagnosed with rheumatoid arthritis (RA) (*n* = 25,903), who had previously been diagnosed with Crohn’s disease (*n* = 1,097), who had previously been diagnosed with ulcerative colitis (UC) (*n* = 1,651), and who had missing variables (*n* = 10,628) before the index date of January 1, 2009. Thus, we included 167,370 eligible participants in the final study population (Supplementary Figure S1).

### Data collection

#### Exposure to antibiotics

The exposure variables considered in this study were the cumulative number of days for antibiotic prescriptions and their respective classes prescribed between 2004 and 2008, analyzed individually. The identification of antibiotic classes was performed using the claim database, followed by classification based on the Anatomical Therapeutic Chemical (ATC) classification of drugs as defined by the World Health Organization (WHO). The categories included macrolides, penicillin, cephalosporin, fluoroquinolones, sulfonamides, lincosamides, tetracyclines, and other classes (Supplementary Table S1) [19, 20]. The antibiotic prescription duration was classified into five categories based on cumulative days: 0, 1–14, 15–30, 31–90, and 91 or more days. Furthermore, the classification of the number of antibiotic classes was designated as 0, 1, 2, 3, and 4 or greater.

#### Definition of osteoporotic fractures

Osteoporotic fractures were identified using specific ICD-10 codes for humerus (ICD-10 codes S422 [upper end of humerus], S423 [shaft of humerus]) and osteoporotic fractures including spine (S22.0 [thoracic spine], S22.1 [multiple fractures of the thoracic spine], S32.0 [lumbar spine], M48.4 [fatigue fracture of vertebra], and M48.5 [collapsed vertebra]); hip (S72.0 [femur neck] and S72.1 [trochanteric fracture]); and radius (S52.5 [lower end of radius] and S52.6 [lower end of both ulnar and radius]) [21]. The ICD-10 codes about fractures related to osteoporosis were obtained from a fact sheet that was published by the Korean Society of Bone and Mineral Research. This allowed us to accurately detect the incidence of osteoporotic fractures within our study cohort [4, 22]. The defined outcome was hospitalization for a minimum of one day or more, or two or more visits to the doctor in 6 months of osteoporotic fractures, whichever event occurred first. Only the initial fracture was regarded as the outcome in cases where multiple fractures were observed during the observation period [22, 23]. Fractures were defined only by the ICD-10 codes mentioned above.

#### Covariates

The analysis was divided into two models to evaluate the impact of various covariates. Model 1 adjusted for age, gender, household income, Charlson comorbidity index (CCI), body mass index (BMI), systolic blood pressure, fasting serum glucose, total cholesterol, smoking status, alcohol intake, and physical activity. Model 2 included all the elements from Model 1 and additionally adjusted for diabetes, calcium and/or vitamin D combination, and steroid use. By including these additional factors in Model 2, we aimed to account for their potential confounding effects on the association between antibiotic use and osteoporotic fracture risk. The index date was the starting point for our follow-up period, with all covariate information obtained before this date. By collecting covariate information before the index date, we aimed to minimize the potential bias in our study and ensure that the exposure variables of cumulative days of prescribing antibiotics and the number of antibiotic classes were assessed in relation to other relevant factors that may have influenced the occurrence of osteoporotic fractures in our study population.

### Statistical analysis

During the follow-up, the primary endpoint was incident osteoporotic fractures. We categorized the exposure to antibiotics according to the antibiotic prescription duration. Cox proportional hazards regression analyses were utilized to ascertain the hazard ratios (HRs) and 95% confidence intervals (CIs) for osteoporotic fracture in antibiotic user groups as compared to antibiotic non-user groups according to cumulative antibiotic prescription days. The association between antibiotic exposure and osteoporotic fracture events was evaluated using the Kaplan–Meier method. We conducted separate calculations for *P* for trend analysis for the total days of antibiotic consumption and the number of antibiotic classes, treating each as a continuous variable. In order to assess for interaction, we incorporated interaction terms between antibiotic consumption and covariates.

We performed multiple sensitivity analyses to verify the association between exposure to antibiotics and incident osteoporotic fractures, according to (1) wash-out periods of 1, 3, or 5 years, (2) an extended exposure period to 6 or 7 years, and (3) a comparison between the group that received non-prescribed antibiotics and the group that was prescribed antibiotics for a specific class only. We also conducted stratified analysis by gender, age, household income, CCI, BMI, diabetes, calcium and/or vitamin D combination, steroid use, and various infectious diseases. Infectious diseases included respiratory diseases, intra-abdominal infections, urinary tract infections (UTI), intestinal infectious diseases, skin, soft tissue, bone, and joint infections (SSTBJ), and other infectious diseases as independent six variables (Supplementary Table S2) [24]. We also evaluated the association between antibiotic exposure and the risk of osteoporotic fracture in individuals with a history of steroid prescriptions and those with a history of diabetes diagnosis, respectively, according to the prescription of calcium and/or vitamin D combinations. The analyses were conducted using SAS Enterprise Guide 7.2 (SAS Institute, USA), and statistical significance was established by a *P* value of less than 0.05. In addition, a Kaplan–Meier curve was produced utilizing R version 3.3.3.

The Seoul National University Hospital Institutional Review Board granted approval for this research (IRB number: E-2204–023-1312), and we did not require informed consent since the NHIS database was created in compliance with strict confidentiality rules after anonymization. This study follows the STROBE (Strengthening Reporting of Observational Studies in Epidemiology) reporting guideline for cohort studies.

## Results

Table 1 and SupplementaryTableS3 present a comprehensive summary of the baseline demographics of those who participated in the study. The research encompassed a cohort of 167,370 participants, with a mean age of 59.3 years and a men’s representation of 60.91%. Among these individuals, a total of 15,170 cases were reported to have developed osteoporotic fractures during the study period. The group that prescribed longer antibiotics exhibited an older age, a higher proportion of women, a higher proportion of diagnosed diabetes, and a higher proportion of prescriptions for calcium and/or vitamin D combination or steroids.

**Table 1:** Baseline characteristics of the study population by the cumulative antibiotic days

Characteristics	Total population	Cumulative days of antibiotics prescribed for 5 years before the index date					*P* value
		None	1–14 days	15–30 days	31–90 days	≥ 91 days	
Number of participants, *n*	167,370	15,283	48,677	39,746	49,892	13,772	
Osteoporotic fracture events, *n*	15,170	1043	3943	3583	5103	1498	
Age, years, mean ± SD	59.3 ± 7.82	58.33 ± 7.39	58.67 ± 7.55	59.11 ± 7.71	59.85 ± 8.01	61.15 ± 8.34	< 0.001
Gender, *n* (%)							< 0.001
Men	101,945 (60.91)	11,236 (73.52)	31,668 (65.06)	23,342 (58.73)	27,551 (55.22)	8148 (59.16)	
Women	65,425 (39.09)	4047 (26.48)	17,009 (34.94)	16,404 (41.27)	22,341 (44.78)	5624 (40.84)	
Household income, *n* (%)							< 0.001
1st quartile (highest)	58,767 (35.11)	5458 (35.71)	17,205 (35.35)	13,941 (35.08)	17,318 (34.71)	4845 (35.18)	
2nd quartile	49,048 (29.31)	4154 (27.18)	14,072 (28.91)	11,653 (29.32)	15,072 (30.21)	4097 (29.75)	
3rd quartile	35,900 (21.45)	3333 (21.81)	10,561 (21.7)	8608 (21.66)	10,505 (21.06)	2893 (21.01)	
4th quartile (lowest)	23,655 (14.13)	2338 (15.3)	6839 (14.05)	5544 (13.95)	6997 (14.02)	1937 (14.06)	
Charlson comorbidity index, *n* (%)							< 0.001
0	32,080 (19.17)	6896 (45.12)	13,562 (27.86)	6327 (15.92)	4570 (9.16)	725 (5.26)	
1	42,410 (25.34)	3840 (25.13)	13,920 (28.6)	11,092 (27.91)	11,334 (22.72)	2224 (16.15)	
≥ 2	92,880 (55.49)	4547 (29.75)	21,195 (43.54)	22,327 (56.17)	33,988 (68.12)	10,823 (78.59)	
Body mass index, kg/m^2^, *n* (%)							< 0.001
BMI < 18.5	3522 (2.1)	456 (2.98)	1123 (2.31)	766 (1.93)	890 (1.78)	287 (2.08)	
18.5 ≤ BMI < 23	57,650 (34.44)	5755 (37.66)	17,601 (36.16)	13,588 (34.19)	16,304 (32.68)	4402 (31.96)	
23 ≤ BMI < 25	47,841 (28.58)	4307 (28.18)	13,782 (28.31)	11,371 (28.61)	14,451 (28.96)	3930 (28.54)	
25 ≤ BMI	58,357 (34.87)	4765 (31.18)	16,171 (33.22)	14,021 (35.28)	18,247 (36.57)	5153 (37.42)	
Systolic blood pressure, mm Hg, mean ± SD	126.81 ± 15.93	128.48 ± 16.8	127.05 ± 16.09	126.51 ± 15.79	126.36 ± 15.68	126.53 ± 15.58	< 0.001
Fasting serum glucose, mg/dL, mean ± SD	100.5 ± 26.73	101.28 ± 27.71	100.54 ± 27.13	100.43 ± 26.65	100.25 ± 26.27	100.55 ± 26.05	0.002
Total cholesterol, mg/dL, mean ± SD	198.71 ± 36.86	197.94 ± 36.45	198.56 ± 36.49	199.17 ± 36.73	198.87 ± 37.14	198.12 ± 37.97	0.001
Smoking status, *n* (%)							< 0.001
Never smoker	118,074 (70.55)	9313 (60.94)	32,960 (67.71)	28,476 (71.64)	37,156 (74.47)	10,169 (73.84)	
Past smoker	17,527 (10.47)	1758 (11.5)	5192 (10.67)	4105 (10.33)	4986 (9.99)	1486 (10.79)	
Current smoker	31,769 (18.98)	4212 (27.56)	10,525 (21.62)	7165 (18.03)	7750 (15.53)	2117 (15.37)	
Alcohol intake, times/week, *n* (%)							< 0.001
0	97,049 (57.98)	7640 (49.99)	26,521 (54.48)	23.202 (58.38)	30,716 (61.56)	8970 (65.13)	
1–2	51,941 (31.03)	5616 (36.75)	16,112 (33.1)	12,224 (30.76)	14,344 (28.75)	3645 (26.47)	
3–4	11,568 (6.91)	1283 (8.39)	3813 (7.83)	2698 (6.79)	3042 (6.1)	732 (5.32)	
≥ 5	6812 (4.07)	744 (4.87)	2231 (4.58)	1622 (4.08)	1790 (3.59)	425 (3.09)	
Physical activity, times/week, *n* (%)							< 0.001
0	75,834 (45.31)	6686 (43.75)	22,168 (45.54)	18,104 (45.55)	22,557 (45.21)	6319 (45.88)	
1–2	47,912 (28.63)	4832 (31.62)	14,317 (29.41)	11,305 (28.44)	13,805 (27.67)	3653 (26.52)	
3–4	23,941 (14.3)	2108 (13.79)	6755 (13.88)	5716 (14.38)	7338 (14.71)	2024 (14.7)	
≥ 5	19,683 (11.76)	1657 (10.84)	5437 (11.17)	4621 (11.63)	6192 (12.41)	1776 (12.9)	
Diabetes, *n* (%)							< 0.001
No	117,106 (69.97)	11,812 (77.29)	35,945 (73.84)	27,905 (70.21)	33,102 (66.35)	8342 (60.57)	
Yes	50,264 (30.03)	3471 (22.71)	12,732 (26.16)	11,841 (29.79)	16,790 (33.65)	5430 (39.43)	
Calcium and/or vitamin D combination, *n* (%)							< 0.001
No	149,735 (89.46)	14,675 (96.02)	45,081 (92.61)	35,580 (89.52)	42,949 (86.08)	11,450 (83.14)	
Yes	17,635 (10.54)	608 (3.98)	3596 (7.39)	4166 (10.48)	6943 (13.92)	2322 (16.86)	
Steroid, *n* (%)							< 0.001
No	46,143 (27.57)	9312 (60.93)	18,392 (37.78)	9855 (24.79)	7422 (14.88)	1162 (8.44)	
Yes	121,227 (72.43)	5971 (39.07)	30,285 (62.22)	29,891 (75.21)	42,470 (85.12)	12,610 (91.56)	

Acronym *n* number of people, *SD* standard deviation

The *P* values were calculated using a *χ*^2^ test for categorical variables and an analysis of variance for continuous variables. The ordering of variables was not considered in the *χ*^2^ test analysis

**Fig. 1:**
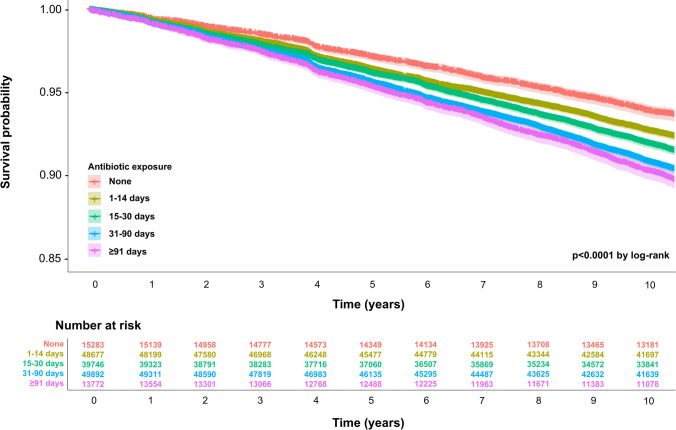
Kaplan–Meier curve of the osteoporotic fracture by the cumulative antibiotic days

The association between the total number of days on antibiotics and the risk of osteoporotic fracture is illustrated in Table 2. According to the multivariable Cox proportional hazards model (Model 2), there was a higher risk of osteoporotic fracture in the group exposed to antibiotics for 91 days or more as compared to those who did not use antibiotics (aHR, 1.12; 95% CI, 1.03–1.21). Further stratification by gender revealed a non-significant association among men (aHR, 1.06; 95% CI, 0.93–1.21) but a significant association among women (aHR, 1.15; 95% CI, 1.03–1.28). Supplementary Table S4 displays the association between the total number of days on antibiotics and the risk of osteoporotic fractures among individuals who were prescribed antibiotics (Model 3). There was a higher risk of osteoporotic fracture in the group exposed to antibiotics for 91 days or more (aHR, 1.06; 95% CI, 1.00–1.13), compared to those who used antibiotics for 1–14 days, after further considering infectious diseases as covariates. Furthermore, the Kaplan–Meier curves of osteoporotic fracture according to antibiotic exposure are illustrated in Fig. 1. In Supplementary Table S5, the association between the cumulative days of antibiotics prescribed and fractures is also presented. The comparison across the entire study population yielded an aHR of 1.09 (95% CI, 1.01–1.18). However, according to Model 2, there was no significant difference when analyzed by gender. This indicates that the association may not be significantly different between men and women when confounding factors are taken into account.

**Table 2:** Association between the cumulative antibiotic days and the risk of osteoporotic fracture

	Cumulative days of antibiotics prescribed for 5 years before the index date					*P* for trend
	None	1–14 days	15–30 days	31–90 days	≥ 91 days	
All						
Number of participants, *n*	15,283	48,677	39,746	49,892	13,772	
Events,* n*	1043	3943	3583	5103	1498	
Person-years	156,238	495,569	403,508	501,633	135,686	
Incidence/10,000 PYs	66.76	79.57	88.80	101.73	110.40	
aHR (95% CI)						
Model 1	1.00 (ref.)	1.06 (0.99–1.14)	1.08 (1.01–1.16)	1.14 (1.06–1.22)	1.17 (1.08–1.27)	< 0.001
Model 2	1.00 (ref.)	1.04 (0.97–1.12)	1.05 (0.97–1.12)	1.09 (1.02–1.17)	1.12 (1.03–1.21)	0.001
Men						
Number of participants, *n*	11,236	31,668	23,342	27,551	8148	
Events, *n*	480	1429	1144	1609	541	
Person-years	115,847	326,320	239,616	279,586	80,607	
Incidence/10,000 PYs	41.43	43.79	47.74	57.55	67.12	
aHR (95% CI)						
Model 1	1.00 (ref.)	0.98 (0.88–1.09)	1.00 (0.90–1.12)	1.10 (0.99–1.22)	1.15 (1.01–1.30)	< 0.001
Model 2	1.00 (ref.)	0.95 (0.85–1.05)	0.95 (0.85–1.06)	1.02 (0.92–1.14)	1.06 (0.93–1.21)	0.05
Women						
Number of participants, *n*	4,047	17,009	16,404	22,341	5624	
Events, *n*	563	2514	2439	3494	957	
Person-years	40,391	169,249	163,892	222,047	55,078	
Incidence/10,000 PYs	139.39	148.54	148.82	157.35	173.75	
aHR (95% CI)						
Model 1	1.00 (ref.)	1.11 (1.02–1.22)	1.12 (1.03–1.23)	1.16 (1.06–1.27)	1.19 (1.07–1.32)	0.001
Model 2	1.00 (ref.)	1.10 (1.00–1.21)	1.10 (1.00–1.21)	1.13 (1.03–1.24)	1.15 (1.03–1.28)	0.024

Acronym *n* number of people, *PY* person-year, *aHR* adjusted hazard ratio, *CI* confidence interval, *ref* reference

The aHRs were calculated by Cox proportional hazards regression after adjustments for multivariate variables. Model 1 adjusted for age, gender, household income, Charlson comorbidity index, body mass index, systolic blood pressure, fasting serum glucose, total cholesterol, smoking status, alcohol intake, and physical activity. Model 2 adjusted for diabetes, calcium and/or vitamin D combination, and steroid in addition to the variables in Model 1

Table 3 displays the sensitivity analysis of the likelihood of osteoporotic fracture based on the cumulative days of antibiotics prescribed. We performed an analysis of the washout period utilizing the 1, 3, or 5-year periods. After being considered a washout period of 1, 3, or 5 years, overall trends were maintained. Analyses were conducted by varying the exposure period to compare the impact of changing the antibiotic exposure period from 5 to 6 or 7 years. After considering 6-year antibiotic exposure, the aHRs of individuals who used antibiotics for 91 days or more were 1.11 (95% CI, 1.02–1.21) compared with non-users and 1.07 (95% CI, 1.01–1.13) compared with people who used antibiotics for 1–14 days. In addition, when considering a 7-year exposure period to antibiotics, the aHRs for individuals who used antibiotics for 91 days or more were 1.15 (95% CI, 1.04–1.26) compared with non-users and 1.08 (95% CI, 1.02–1.14) compared with people who used antibiotics for 1–14 days. It may be confirmed that similar significant associations were observed across all three different exposure periods.

**Table 3:** Sensitivity analysis of the association between the cumulative antibiotic days and the risk of osteoporotic fracture

aHR (95% CI)	Cumulative days of antibiotics prescribed for 5 years before the index date							*P* for trend
	Total	Events	None	1–14 days	15–30 days	31–90 days	** ≥ **91 days	
Wash-out period								
Model ^a^								
1-year wash-out	166,347	14,147	1.00 (ref.)	1.04 (0.97–1.12)	1.05 (0.97–1.12)	1.09 (1.02–1.18)	1.12 (1.03–1.22)	0.001
3-year wash-out	163,760	11,560	1.00 (ref.)	1.02 (0.94–1.10)	1.03 (0.95–1.12)	1.08 (0.99–1.16)	1.13 (1.03–1.24)	0.001
5-year wash-out	160,739	8539	1.00 (ref.)	0.99 (0.91–1.09)	1.04 (0.95–1.14)	1.08 (0.99–1.19)	1.13 (1.01–1.26)	< 0.001
Model ^b^								
1-year wash-out	151,133	13,173		1.00 (ref.)	1.00 (0.96–1.05)	1.05 (1.01–1.10)	1.08 (1.01–1.15)	0.004
3-year wash-out	148,704	10,744		1.00 (ref.)	1.01 (0.96–1.07)	1.06 (1.01–1.11)	1.11 (1.03–1.19)	0.001
5-year wash-out	145,894	7934		1.00 (ref.)	1.05 (0.99–1.11)	1.09 (1.03–1.16)	1.14 (1.05–1.24)	< 0.001
Variation of the exposure period								
6-year exposure period								
Model^a^	167,370	15,170	1.00 (ref.)	1.04 (0.96–1.12)	1.07 (0.99–1.15)	1.07 (0.99–1.15)	1.11 (1.02–1.21)	0.014
Model^b^	155,112	14,352		1.00 (ref.)	1.03 (0.98–1.08)	1.03 (0.99–1.08)	1.07 (1.01–1.13)	0.042
7-year exposure period								
Model^a^	167,370	15,170	1.00 (ref.)	1.06 (0.97–1.16)	1.09 (1.00–1.19)	1.09 (1.00–1.19)	1.15 (1.04–1.26)	0.004
Model^b^	155,112	14,352		1.00 (ref.)	1.03 (0.98–1.08)	1.03 (0.98–1.08)	1.08 (1.02–1.14)	0.022

Acronym *aHR* adjusted hazard ratio, *CI* confidence interval, *ref* reference

^a^Antibiotics non-user group was set as a reference group

^b^Antibiotics 1–14 days user group was set as a reference group

The aHRs were calculated by Cox proportional hazards regression after adjustments for multivariate variables. Model adjusted for age, gender, household income, Charlson comorbidity index, body mass index, systolic blood pressure, fasting serum glucose, total cholesterol, smoking status, alcohol intake, physical activity, diabetes, calcium and/or vitamin D combination, and steroid

The antibiotic exposure period in the main analyses was set from 2004 to 2008, a total of 5 years. The 6-year exposure period in sensitivity analyses indicates that the antibiotic exposure period was set from 2003 to 2008, a total of 6 years. The 7-year exposure period in sensitivity analyses indicates that the antibiotic exposure period was set from 2002 to 2008, a total of 7 years

The subgroup analyses stratified by major variables are presented in Supplementary Table S6. Statistically significant interactions were not found in most of the subgroups, including five variables of infectious diseases, except for age and UTI. In the group of individuals over the age of 60 or who had no history of UTI, respectively, long-term antibiotic exposure was associated with an increased risk of osteoporotic fracture. The stratified analysis within the steroid prescription group, as presented in Supplementary Table S7, compares the risk of osteoporotic fractures according to their cumulative antibiotic exposure days between those prescribed calcium and/or vitamin D combinations and those not. Among participants not prescribing calcium and/or vitamin D combinations, those with ≥ 91 days of antibiotic exposure had an aHR of 1.08 (95% CI, 0.96–1.21). In contrast, among participants prescribing calcium and/or vitamin D combinations, those with ≥ 91 days of antibiotic exposure had an aHR of 0.95 (95% CI, 0.71–1.27). Among individuals with a history of steroid prescriptions, there was a trend toward an increased risk of osteoporotic fracture with higher cumulative days of antibiotic prescriptions among participants without a history of calcium and/or vitamin D combinations, but the values were not statistically significant. Additionally, Supplementary Table S8 examines the association between the cumulative antibiotic days and the risk of osteoporotic fracture among individuals who have been diagnosed with diabetes, according to the prescription of calcium and/or vitamin D combinations. When calcium and/or vitamin D combinations were not prescribed, the aHR was 1.06 (95% CI, 0.91–1.24). When these medications were prescribed, the aHR was 1.37 (95% CI, 0.88–2.15). These findings indicate that, even among individuals diagnosed with diabetes, there was no statistically significant association. In patients with diabetes, there was a trend toward an increased risk of osteoporotic fracture with increasing cumulative days of antibiotic prescriptions, with or without prescription of calcium and/or vitamin D combinations, but the values were not statistically significant.

The relationships between the prevalence of osteoporotic fractures and the number of antibiotic class categories are shown in Table 4. According to Model 2, individuals who utilized more than four antibiotic classes exhibited an increased likelihood of osteoporotic fracture (aHR, 1.10; 95% CI, 1.02–1.18) in comparison to those who did not prescribe antibiotics. The association between the number of antibiotic classes and osteoporotic fractures among individuals who were prescribed antibiotics is detailed in Supplementary Table S9. After further considering infectious diseases as covariates (Model 3), individuals who prescribed more than four antibiotic classes exhibited an increased osteoporotic fracture risk (aHR, 1.06; 95% CI, 1.00–1.12) in comparison to those who only used one class of antibiotics. When comparing the antibiotic non-user group to the group prescribed only one certain antibiotic class during the exposure period, no specific antibiotic class was statistically significantly associated with an increased risk of osteoporotic fracture (Supplementary Table S10).

**Table 4:** Association between the number of prescribed antibiotic classes and the risk of osteoporotic fracture

	Number of antibiotic classes prescribed during 5 years before the index date					*P* for trend
	None	1	2	3	≥ 4	
All						
Number of participants, *n*	15,283	27,699	41,823	47,018	35,547	
Events,* n*	1043	2205	3790	4428	3704	
Person-years	156,238	281,193	423,162	474,609	357,431	
aHR (95% CI)						
Model 1	1.00 (ref.)	1.05 (0.97–1.13)	1.11 (1.03–1.19)	1.09 (1.02–1.17)	1.16 (1.08–1.24)	< 0.001
Model 2	1.00 (ref.)	1.03 (0.95–1.11)	1.07 (0.95–1.11)	1.05 (0.98–1.12)	1.10 (1.02–1.18)	0.01
Men						
Number of participants, *n*	11,236	18,486	25,939	26,991	19,293	
Events, *n*	480	826	1355	1428	1114	
Person-years	115,847	189,753	265,152	275,196	196,027	
aHR (95% CI)						
Model 1	1.00 (ref.)	0.96 (0.86–1.08)	1.06 (0.96–1.18)	1.03 (0.92–1.14)	1.08 (0.97–1.21)	0.051
Model 2	1.00 (ref.)	0.93 (0.83–1.05)	1.01 (0.91–1.12)	0.96 (0.86–1.07)	1.00 (0.89–1.12)	0.778
Women						
Number of participants, *n*	4047	9213	15,884	20,027	16,254	
Events, *n*	563	1379	2435	3000	2590	
Person-years	40,391	91,440	158,010	199,413	161,403	
aHR (95% CI)						
Model 1	1.00 (ref.)	1.10 (1.00–1.22)	1.13 (1.03–1.24)	1.12 (1.03–1.23)	1.20 (1.09–1.31)	< 0.001
Model 2	1.00 (ref.)	1.09 (0.99–1.20)	1.11 (1.01–1.22)	1.10 (1.00–1.20)	1.16 (1.06–1.28)	0.007

Acronym *n* number of people, *SD* standard deviation

The aHRs were calculated by Cox proportional hazards regression after adjustments for multivariate variables. Model 1 adjusted for age, gender, household income, Charlson comorbidity index, body mass index, systolic blood pressure, fasting serum glucose, total cholesterol, smoking status, alcohol intake, and physical activity. Model 2 adjusted for diabetes, calcium and/or vitamin D combination, and steroid in addition to the variables in Model 1

Antibiotics were divided into eleven classes consisting of penicillin, cephalosporin, macrolide, fluoroquinolone, sulfonamides, tetracyclines, and lincosamides or others

## Discussion

Our population-based cohort study found a significant association between antibiotic use and osteoporotic fractures in individuals aged 50 years and older in Korea. An association between the incident osteoporotic fractures and the cumulative duration of antibiotic exposure was observed. These associations remained significant after adjusting for various covariates. As the number of classes used increased, the possibility of osteoporotic fracture incidence also increased. To assess the association between antibiotic exposure and the risk of osteoporotic fractures more clearly, we excluded individuals diagnosed with conditions such as RA, Crohn’s disease, and UC, or those prescribed specific medications such as bisphosphonates, which could potentially influence the assessment of this association. The reliability and validity of our findings were also enhanced through the implementation of multiple sensitivity analyses.

Specifically, the stratified analysis by prescription of calcium and/or vitamin D combination, which are related to osteoporotic patients, showed that the risk of osteoporotic fracture was statistically significantly higher with longer days of antibiotic prescription only in the group not prescribed calcium and/or vitamin D combination. Conversely, the risk of osteoporotic fracture did not statistically significantly increase with the cumulative number of antibiotic prescription days in the group of individuals with a prescription history of steroids, which could increase the risk of infectious diseases and fractures.

Furthermore, the analysis using Model 2, which includes factors such as steroid use and diabetes, indicates that while there is a significant overall association between antibiotic use and fracture risk, this association does not significantly differ when analyzed separately by gender. This ensures that the potential confounding effects of steroid use and diabetes are accounted for, providing a more accurate assessment of the relationship between antibiotic use and fracture risk. Specifically, our findings show that in women, long-term antibiotic use is significantly associated with an increased risk of osteoporotic fractures (*P* for trend 0.024), which may be due to decreased estrogen levels post-menopause [25]. In contrast, for men, the association is significant for total fractures (*P* for trend 0.03) but not for osteoporotic fractures (*P* for trend 0.05). These differences may suggest the possibility of a gender-specific hormonal change in overall fracture risk.

Although an association between antibiotic use and fractures has been demonstrated, the exact mechanisms underlying this association remain incompletely understood. According to certain studies, prolonged utilization of antibiotics may potentially modify the gut microbiota and reduce bone density, thereby increasing the likelihood of fractures [26, 27]. A recent study has revealed that the gut microbiota is a complex network of metabolically interdependent microorganisms [28, 29]. The symbiotic gut microbiota plays a crucial role in facilitating digestion, regulating and stimulating the immune system, and inhibiting the proliferation of pathogens [30]. The increasing prevalence of antibiotic usage is a matter of great concern due to its potential to disrupt the gut microbiota [13]. Furthermore, specific categories of antibiotics may have been associated with reduced levels of vitamin K2 [31], which plays a crucial role in preserving bone health [32, 33]. The occurrence of vitamin K deficiency is primarily attributed to the utilization of specific medications such as certain antibiotics, VK antagonist anticoagulants, and anticonvulsants [34].

Additionally, liver and pancreatic diseases may also contribute to this deficiency [35]. A non-collagenous protein called osteocalcin is released by osteoblasts and has three glutamate residues that are amenable to carboxylation [36]. The aforementioned alteration is facilitated by γ-glutamyl carboxylase, which relies on vitamin K, O_2_, and CO_2_ as cofactors, obtained through the vitamin K cycle and circulation [37]. Carboxylated osteocalcin is a crucial factor in achieving the proper alignment of apatite crystals and promoting optimal bone strength [38]. Antibiotics have been observed to disrupt estrogen metabolism, resulting in a decrease in the circulating levels of estriol [39]. Osteoporosis is a condition characterized by the progression of bone resorption resulting from estrogen deficiency and reduced bone density [40, 41]. Osteoporosis is commonly recognized as a significant risk factor for fractures [41]. Fractures associated with osteoporosis, including those affecting the vertebral body, forearm, and proximal femur, are more likely to occur [15].

This study has certain limitations. Firstly, the reliance on a database solely from South Korea raises the possibility of potential biases, including regional and cultural biases. Therefore, our findings may lack generalizability to other nationalities. Secondly, selection bias may have occurred, given that the individuals who underwent health examinations may have been healthier than non-participants, even though the study population was randomly chosen from the national health examination-based cohort. Thirdly, the NHIS dataset lacks imaging records, including X-rays, which are necessary to substantiate the operational definition of osteoporotic fracture. Furthermore, the NHIS database lacks data on bone mineral density (BMD), fall history, and dietary habits. Future investigations incorporating BMD measurements are imperative for gaining an extensive understanding of the association between antibiotic use and osteoporotic fractures. Additional trials that take into account fracture risk factors as confounding variables are necessary. Fourthly, exposure to four or more classes of antibiotics or long-term antibiotic exposure may represent individuals with more chronic infections or severe illnesses, potentially limiting the generalizability of our findings. Even though we excluded individuals who had a history of RA, Crohn’s disease, UC, or bisphosphonate medication and performed several statistical analyses considering various covariates, the potential for bias still exists. Therefore, to assess the association more accurately between long-term antibiotic exposure and the risk of osteoporosis fractures, it is necessary to consider the distribution of characteristics among antibiotic non-users and long-term prescription groups through methodologies such as propensity score matching. Studies have shown that even short-term exposure to antibiotics, such as 7 days, can cause an imbalance in the gut microbiome [42, 43], and that recovery from antibiotic exposure could take up to 4 years [44] or not recover [45]. While these findings suggest that an exposure variable of cumulative antibiotic prescription days over a period may be one suitable operational definition, to better assess the impact of antibiotic exposure on osteoporotic fractures, further studies that follow up osteoporotic fracture occurrence from the number of days since the last antibiotic prescription are needed, as well as experimental studies that can identify the actual gut dysbiosis associated with antibiotic exposure. Furthermore, there is a potential for reverse causation. Osteoporotic fractures may result in reduced physical activity, leading to compromised immunity and heightened susceptibility to infections. Consequently, patients with osteoporotic fractures may require antibiotics to manage infections. In order to address this limitation, our sensitivity analysis incorporated washout periods and varying exposure periods. Lastly, due to the retrospective nature, it was not possible to completely eliminate the potential for indication bias. Finally, as an observational study, a causal relationship cannot be demonstrated with this retrospective cohort study.

Despite these limitations, the study’s strengths lie in its capacity to evaluate the association between antibiotic exposure and osteoporotic fractures among adults using the valuable NHIS database, which features representative population-based cohort data. Also, multiple analyses were performed, considering various covariates, enhancing the study’s reliability and robustness. These strengths contribute valuable insights into the potential relationship between antibiotic use and osteoporotic fractures.

## Conclusion/summary

In conclusion, this extensive cohort study in Korea revealed a significant association between prolonged antibiotic use and an increased risk of osteoporotic fractures. This underscores the need for caution in prescribing antibiotics, particularly for individuals with fragile bone health. Further research is required to elucidate the underlying mechanisms and refine clinical recommendations.

## Supplementary Information

Below is the link to the electronic supplementary material.

ESM1 (DOCX 338 KB)

## Data Availability

The data utilized in this study were provided by the Korean National Health Insurance Service (KNHIS). Access to thisdatabase is restricted and requires approval from the KNHIS. Only authorized researchers are permitted to accessthe database. Due to privacy and confidentiality concerns, the data are not publicly available.

## Declarations

### Prior presentations

None exist.
