# Supplementary material for: Association between antibiotics use and osteoporotic fracture risk: a nationally representative retrospective cohort study
Source: Arch Osteoporos. 2024 Aug 30;19(1):81. doi: 10.1007/s11657-024-01438-8 (PMC11364706; doi:10.1007/s11657-024-01438-8)
Supplement: Supplementary file 1 — (DOCX 338 KB) [file 11657_2024_1438_MOESM1_ESM.docx]

**Supplementary Materials**

**Association between antibiotics and osteoporotic fracture risk: a nationally representative retrospective cohort study**

Ji Won Lee, BS^1,2,^**^†^**, Sun Jae Park, BS^1,^**^†^**, Young Jun Park, MD^3^, Seogsong Jeong, MD, PhD^4^, Jihun Song, MS^1^, Hye Jun Kim, MS^1^, Jooyoung Chang, MD, PhD^1^, Kyae Hyung Kim, MD, PhD^5,6^, Ji Soo Kim, MD, PhD^7^, Yun Hwan Oh, MD, PhD^8^, Yoosun Cho, MD, PhD^9^ and Sang Min Park, MD, PhD^1,5^

^1^Department of Biomedical Sciences, Seoul National University Hospital, Seoul National University College of Medicine, Seoul, South Korea

^2^Department of Health Convergence, Ewha Womans University, Seoul, South Korea

^3^Medical Research Center, Genomic Medicine Institute, Seoul National University, Seoul, South Korea

^4^Department of Biomedical Informatics, Korea University College of Medicine, Seoul, South Korea

^5^Department of Family Medicine, Seoul National University Hospital, Seoul National University College of Medicine, Seoul, South Korea

^6^Comprehensive Care Clinic, Public Healthcare Center, Seoul National University Hospital, Seoul, South Korea

^7^International Healthcare Center, Seoul National University Bundang Hospital, Seongnam, South Korea

^8^Department of Family medicine, Chung-Ang University Gwangmyeong Hospital, Chung-Ang University College of Medicine, Gwangmyeong-si, South Korea

^9^Total Healthcare Center, Kangbuk Samsung Hospital, Sungkyunkwan University School of Medicine, Seoul, South Korea

**^†^**These authors have contributed equally to this work and share the first authorship.

**Correspondence:** Sang Min Park

Sang Min Park, Department of Biomedical Sciences and Family Medicine, Seoul National University Hospital, Seoul National University College of Medicine, 101 Daehak-ro, Jongno-gu, Seoul, South Korea

**Phone**: 82-2-2072-3331, **Fax**: 82-2-766-3276

**E-mail**: smpark.snuh@gmail.com

**ORCiD**: 0000-0002-7498-4829

**Supplementary Figure S1. Selection of cohort study participants.**

**Supplementary Table S1. A list of antibiotics for each class based on guidelines by the World Health Organization Anatomical Therapeutic Chemical (WHO ATC).**

**Supplementary Table S2. ICD-10 codes for representative sources of infection, organized by systems.**

**Supplementary Table S3. Baseline characteristics of the study population by the cumulative antibiotic days for infectious disease.**

**Supplementary Table S4. Association between the cumulative antibiotic days and the risk of osteoporotic fracture among participants who were prescribed antibiotics.**

**Supplementary Table S5. Association between the cumulative antibiotic days and the risk of fracture.**

**Supplementary Table S6. Stratified analysis of the association between the cumulative antibiotic days and the risk of osteoporotic fracture.**

**Supplementary Table S7. Stratified analysis of the association between the cumulative antibiotic days and the risk of osteoporotic fracture according to the prescription of calcium and/or vitamin D combinations among individuals who have been prescribed steroids.**

**Supplementary Table S8. Stratified analysis of the association between the cumulative antibiotic days and the risk of osteoporotic fracture according to the prescription of calcium and/or vitamin D combinations among individuals who have been diagnosed with diabetes.**

**Supplementary Table S9. Association between the number of prescribed antibiotic classes and the risk of osteoporotic fracture among participants who were prescribed antibiotics.**

**Supplementary Table S10. Comparison of specific antibiotic class users and antibiotic non-users for osteoporotic fracture risk.**

**Supplementary Figure S1. Selection of cohort study participants.**

**
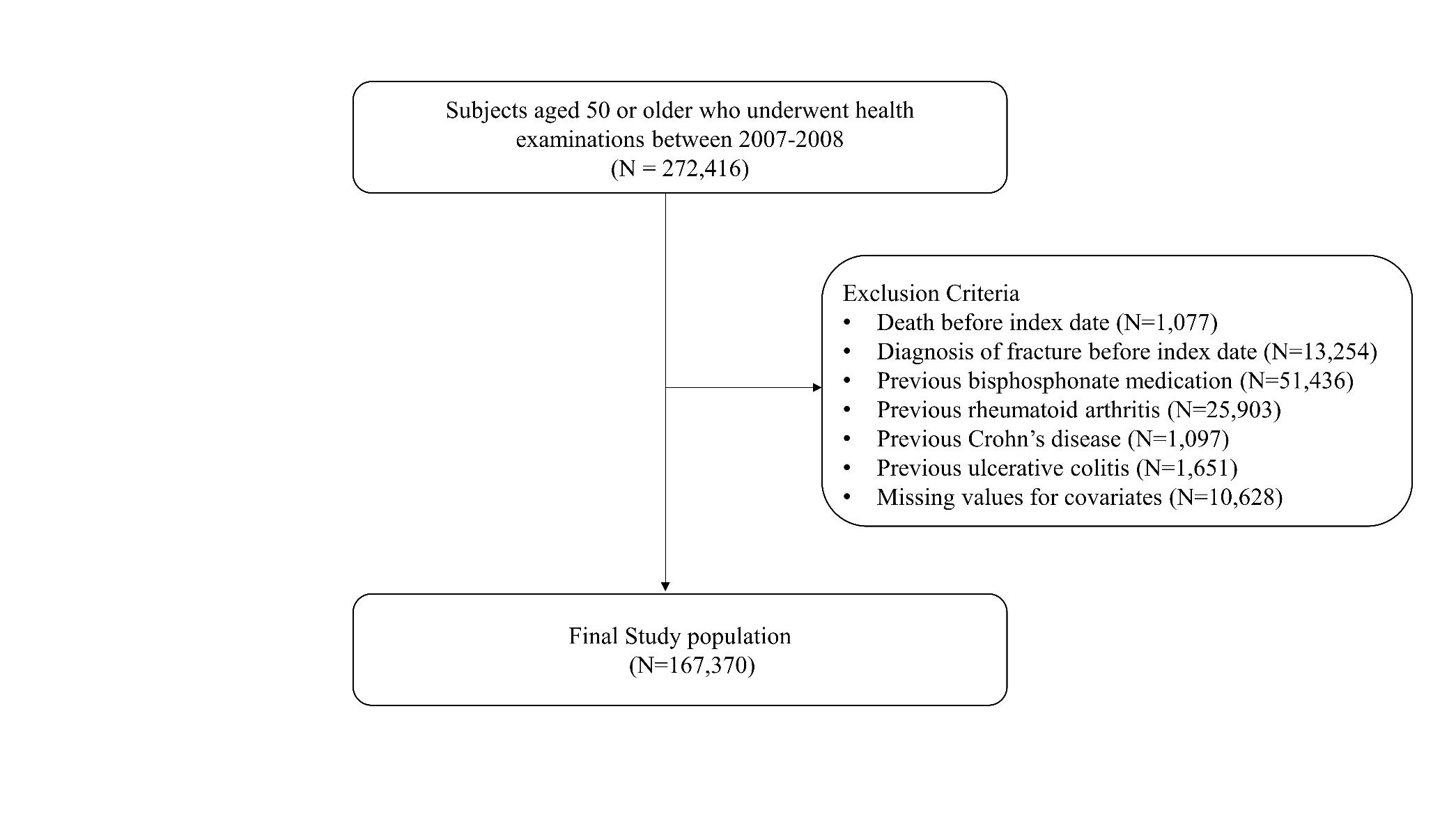
**

**Supplementary Table S1. A list of antibiotics for each class based on guidelines by the World Health Organization Anatomical Therapeutic Chemical (WHO ATC).**

| **Antibiotics class** | **Types of antibiotics for each class** |
| --- | --- |
| Tetracyclines | doxycycline, tetracycline |
| Macrolides | erythromycin, spiramycin, midecamycin, oleandomycin, roxithromycin, josamycin, troleandomycin, clarithromycin, azithromycin, miocamycin, rokitamycin, dirithromycin, flurithromycin, telithromycin, solithromycin |
| Fluoroquinolones | ofloxacin, ciprofloxacin, pefloxacin, enoxacin, temafloxacin, norfloxacin, levofloxacin, moxifloxacin, gemifloxacin, gatifloxacin, sitafloxacin |
| Penicillins | ampicillin, pivamicillin, carbenicillin, amoxicillin, amoxicillin and clavulanate, azlocillin, mezlocillin, mecillinam, piperacillin, ticarcillin, metampicillin, talampicillin, dicloxacillin, oxacillin |
| Sulfonamides | sulfaisodimidine, sulfamethizole, sulfadimidine, sulfapyridine, sulfafurazole, sulfanilamide, sulfathiazole, sulfathiourea, sulfamethoxazole and trimethoprim, sulfadiazine, sulfasalazine, sulfamoxole |
| Lincosamides | clindamycin, lincomycin |
| Vancomycin | vancomycin |
| Carbapenems | meropenem, ertapenem |
| Cephalosporins | cefalexin, cefaloridine, cefalotin, cefazolin, cefatrizine, ceftezole, cefoxitin, cefotetan, cefonicid, cefotaxime, ceftazidime, ceftriaxone, cefmenoxime, cefdinir, cefteram, cefepime, cefpirome, cefaclor, cefuroxime |
| Monobactams | aztreonam, carumonam |
| Linezolid | linezolid |

**Supplementary Table S2. ICD-10 codes for representative sources of infection, organized by systems.**

| **Infectious diseases** | **ICD-10 codes** |
| --- | --- |
| **Respiratory diseases** |  |
| Acute upper respiratory infections | J00, J01, J02, J03, J04, J05, J06 |
| Pneumonia & influenza | J09, J10, J11, J12, J13, J14, J15, J16, J17, J18 |
| Chronic bronchitis | J41, J42 |
| **Intra-abdominal infections** |  |
| Cholecystitis & cholangitis | K80, K81, K83 |
| Appendicitis | K35, K36, K37 |
| Diverticulitis | K57 |
| Peritonitis | K65 |
| Pancreatitis | K85 |
| **Urinary tract infections** |  |
| Cystitis | N30 |
| Acute pyelonephritis | N10 |
| Urethritis | N34, N37 |
| **Intestinal infectious diseases** | A00, A01, A02, A03, A04, A05, A06, A07, A08, A09 |
| **Skin, soft tissue, bone and joint infections** |  |
| Cellulitis | L03 |
| Erysipelas | A46 |
| Impetigo | L01 |
| Folliculitis | L66.2, L66.4 |
| Furuncle & carbuncle | L02 |
| Osteomyelitis | M86 |
| Synovitis | M65, M67, M68, M70 |
| **Other infectious diseases** |  |
| Acute/chronic otitis media | H65, H66 |
| Sepsis | A40, A41 |
| Central nervous system infection | A81, A89 |

Acronym: ICD-10, International Classification of Diseases, Tenth Revision.

**Supplementary Table S3. Baseline characteristics of the study population by the cumulative antibiotic days for infectious disease.**

| Characteristics | Total  population | Cumulative days of antibiotics prescribed for 5 years before the index date | | | | | *P* value |
| --- | --- | --- | --- | --- | --- | --- | --- |
|  |  | None | 1-14 days | 15-30 days | 31-90 days | ≥91days |  |
| Number of participants, n | 167,370 | 15,283 | 48,677 | 39,746 | 49,892 | 13,772 |  |
| Osteoporotic fracture events, n | 15,170 | 1,043 | 3,943 | 3,583 | 5,103 | 1,498 |  |
| Respiratory diseases, n (%) | | | | | | | <0.001 |
| No | 27,184 (16.24) | 8,169 (53.45) | 11,170 (22.95) | 4,466 (11.24) | 2,879 (5.77) | 500 (3.63) |  |
| Yes | 140,186 (83.76) | 7,114 (46.55) | 37,507 (77.05) | 35,280 (88.76) | 47,013 (94.23) | 13,272 (96.37) |  |
| Intra-abdominal infections, n (%) | | | | | | | <0.001 |
| No | 160,555 (95.93) | 14,924 (97.65) | 47,063 (96.68) | 38,082 (95.81) | 47,459 (95.12) | 13,027 (94.59) |  |
| Yes | 6,815 (4.07) | 359 (2.35) | 1,614 (3.32) | 1,664 (4.19) | 2,433 (4.88) | 745 (5.41) |  |
| Urinary tract infections, n (%) | | | | | | | <0.001 |
| No | 139,777 (83.51) | 14,711 (96.26) | 44,264 (90.93) | 33,461 (84.19) | 37,800 (75.76) | 9,541 (69.28) |  |
| Yes | 27,593 (16.49) | 572 (3.74) | 4,413 (9.07) | 6,285 (15.81) | 12,092 (24.24) | 4,231 (30.72) |  |
| Intestinal infectious diseases, n (%) | | | | | | | <0.001 |
| No | 135,091 (80.71) | 14,307 (93.61) | 41,872 (86.02) | 32,065 (80.67) | 37,190 (74.54) | 9,657 (70.12) |  |
| Yes | 32,279 (19.29) | 976 (6.39) | 6,805 (13.98) | 7,681 (19.33) | 12,702 (25.46) | 4,115 (29.88) |  |
| Skin, soft tissue, bone, and joint infections, n (%) | | | | | | | <0.001 |
| No | 77,771 (46.47) | 10,671 (69.82) | 26,725 (54.9) | 17,723 (44.59) | 18,295 (36.67) | 4,357 (31.64) |  |
| Yes | 89,599 (53.53) | 4,612 (30.18) | 21,952 (45.1) | 22,023 (55.41) | 31,597 (63.33) | 9,415 (68.36) |  |
| Other infectious diseases, n (%) | | | | | | | <0.001 |
| No | 149,871 (89.54) | 14,971 (97.96) | 46,010 (94.52) | 36,064 (90.74) | 42,363 (84.91) | 10,463 (75.97) |  |
| Yes | 17,499 (10.46) | 312 (2.04) | 2,667 (5.48) | 3,682 (9.26) | 7,529 (15.09) | 3,309 (24.03) |  |

The *P* values were calculated using a χ^2^ test for categorical variables. The ordering of variables was not considered in the χ^2^ test analysis.

Acronym: n, number of people.

**Supplementary Table S4. Association between the cumulative antibiotic days and the risk of osteoporotic fracture among participants who were prescribed antibiotics.**

|  | Cumulative days of antibiotics prescribed for 5 years before the index date | | | | *P* for trend |
| --- | --- | --- | --- | --- | --- |
|  | 1-14 days | 15-30 days | 31-90 days | ≥91days |  |
| Number of participants, n | 48,677 | 39,746 | 49,892 | 13,772 |  |
| Events, n | 3,943 | 3,583 | 5,103 | 1,498 |  |
| Person-years | 495,569 | 403,508 | 501,633 | 135,686 |  |
| aHR (95% CI) |  |  |  |  |  |
| Model 1 | 1.00 (ref.) | 1.02 (0.97-1.07) | 1.07 (1.03-1.12) | 1.10 (1.04-1.17) | <0.001 |
| Model 2 | 1.00 (ref.) | 1.01 (0.96-1.05) | 1.05 (1.01-1.10) | 1.08 (1.01-1.14) | 0.005 |
| Model 3 | 1.00 (ref.) | 1.00 (0.96-1.05) | 1.04 (0.99-1.09) | 1.06 (1.00-1.13) | 0.029 |

The aHRs were calculated by Cox proportional hazards regression after adjustments for multivariate variables. Model 1 adjusted for age, gender, household income, Charlson comorbidity index, body mass index, systolic blood pressure, fasting serum glucose, total cholesterol, smoking status, alcohol intake, and physical activity. Model 2 adjusted for diabetes, calcium and/or vitamin D combination, and steroid in addition to the variables in Model 1. Model 3 adjusted for infectious diseases (respiratory diseases, intra-abdominal infections, urinary tract infections, intestinal infectious diseases, skin, soft tissue, bone, and joint infections, and other infectious diseases) in addition to the variables in Model 2.

Acronym: n, number of people; aHR, adjusted hazard ratio; CI, confidence interval; ref, reference.

**Supplementary Table S5. Association between the cumulative antibiotic days and the risk of fracture.**

|  | Cumulative days of antibiotics prescribed for 5 years before the index date | | | | | *P* for trend |
| --- | --- | --- | --- | --- | --- | --- |
|  | None | 1-14 days | 15-30 days | 31-90 days | ≥91days |  |
| All |  |  |  |  |  |  |
| Number of participants, n | 15,283 | 48,677 | 39,746 | 49,892 | 13,772 |  |
| Events, n | 1,200 | 4,514 | 4,076 | 5,757 | 1,714 |  |
| Person-years | 161,213 | 515,005 | 421,113 | 526,499 | 142,730 |  |
| Incidence/10,000 PYs | 74.44 | 87.65 | 96.79 | 109.34 | 120.09 |  |
| aHR (95% CI) | | | | | | |
| Model 1 | 1.00 (ref.) | 1.04 (0.97-1.11) | 1.05 (0.98-1.12) | 1.09 (1.03-1.17) | 1.14 (1.06-1.23) | <0.001 |
| Model 2 | 1.00 (ref.) | 1.02 (0.95-1.09) | 1.02 (0.95-1.09) | 1.05 (0.98-1.12) | 1.09 (1.01-1.18) | 0.008 |
| Men |  |  |  |  |  |  |
| Number of participants, n | 11,236 | 31,668 | 23,342 | 27,551 | 8,148 |  |
| Events, n | 562 | 1,716 | 1,362 | 1,917 | 654 |  |
| Person-years | 118,036 | 332,772 | 244,791 | 286,759 | 82,810 |  |
| Incidence/10,000 PYs | 47.61 | 51.57 | 55.64 | 66.85 | 78.98 |  |
| aHR (95% CI) |  |  |  |  |  |  |
| Model 1 | 1.00 (ref.) | 1.00 (0.91-1.10) | 1.00 (0.91-1.11) | 1.10 (1.00-1.21) | 1.18 (1.05-1.33) | <0.001 |
| Model 2 | 1.00 (ref.) | 0.96 (0.87-1.06) | 0.95 (0.85-1.05) | 1.02 (0.92-1.13) | 1.08 (0.96-1.22) | 0.030 |
| Women |  |  |  |  |  |  |
| Number of participants, n | 4,047 | 17,009 | 16,404 | 22,341 | 5,624 |  |
| Events, n | 638 | 2,798 | 2,714 | 3,840 | 1,060 |  |
| Person-years | 43,176 | 182,232 | 176,321 | 239,740 | 59,919 |  |
| Incidence/10,000 PYs | 147.77 | 153.54 | 153.92 | 160.17 | 176.91 |  |
| aHR (95% CI) |  |  |  |  |  |  |
| Model 1 | 1.00 (ref.) | 1.05 (0.96-1.14) | 1.05 (0.97-1.15) | 1.07 (0.98-1.16) | 1.09 (0.99-1.21) | 0.086 |
| Model 2 | 1.00 (ref.) | 1.04 (0.95-1.13) | 1.04 (0.95-1.13) | 1.05 (0.96-1.14) | 1.06 (0.96-1.18) | 0.302 |

The aHRs were calculated by Cox proportional hazards regression after adjustments for multivariate variables. Model 1 adjusted for age, gender, household income, Charlson comorbidity index, body mass index, systolic blood pressure, fasting serum glucose, total cholesterol, smoking status, alcohol intake, and physical activity. Model 2 adjusted for diabetes, calcium and/or vitamin D combination, and steroid in addition to the variables in Model 1.

Acronym: n, number of people; PY, person-year; aHR, adjusted hazard ratio; CI, confidence interval; ref, reference.

**Supplementary Table S6. Stratified analysis of the association between the cumulative antibiotic days and the risk of osteoporotic fracture.**

| aHR (95% CI) | Total | Events | Cumulative days of antibiotics prescribed for 5 years before the index date | | | | | | | | *P* for trend | *P* for interaction |
| --- | --- | --- | --- | --- | --- | --- | --- | --- | --- | --- | --- | --- |
|  |  |  | None | 1-14 days | | 15-30 days | | 31-90 days | | ≥91days |  |  |
| Age |  |  |  |  |  | |  | |  | |  | 0.024 |
| 50-59 years | 99,501 | 5,822 | 1.00 (ref.) | 0.98 (0.88-1.08) | 0.97 (0.87-1.08) | | 0.98 (0.88-1.09) | | 1.01 (0.88-1.16) | | 0.854 |  |
| ≥60 years | 67,869 | 9,348 | 1.00 (ref.) | 1.07 (0.98-1.18) | 1.08 (0.99-1.19) | | 1.15 (1.05-1.26) | | 1.20 (1.08-1.33) | | <0.001 |  |
| Household income |  |  |  |  |  | |  | |  | |  | 0.422 |
| High | 107,815 | 9,222 | 1.00 (ref.) | 0.98 (0.90-1.07) | 0.99 (0.90-1.08) | | 1.02 (0.93-1.12) | | 1.05 (0.95-1.17) | | 0.079 |  |
| Low | 59,555 | 5,948 | 1.00 (ref.) | 1.14 (1.02-1.27) | 1.14 (1.02-1.28) | | 1.20 (1.07-1.35) | | 1.22 (1.07-1.40) | | 0.003 |  |
| Charlson comorbidity index |  |  |  |  |  | |  | |  | |  | 0.172 |
| 0 | 32,080 | 2,135 | 1.00 (ref.) | 1.12 (0.99-1.26) | 1.13 (0.98-1.30) | | 1.01 (0.87-1.18) | | 0.86 (0.62-1.20) | | 0.667 |  |
| ≥1 | 135,290 | 13,035 | 1.00 (ref.) | 1.00 (0.92-1.09) | 1.02 (0.93-1.11) | | 1.09 (1.00-1.18) | | 1.12 (1.02-1.23) | | <0.001 |  |
| Body mass index |  |  |  |  |  | |  | |  | |  | 0.602 |
| <25 kg/m^2^ | 109,013 | 10,246 | 1.00 (ref.) | 1.03 (0.95-1.12) | 1.04 (0.95-1.13) | | 1.06 (0.98-1.16) | | 1.12 (1.01-1.24) | | 0.021 |  |
| ≥25 kg/m^2^ | 58,357 | 4,924 | 1.00 (ref.) | 1.05 (0.92-1.20) | 1.05 (0.92-1.21) | | 1.13 (0.99-1.29) | | 1.10 (0.94-1.28) | | 0.049 |  |
| Diabetes |  |  |  |  |  | |  | |  | |  | 0.218 |
| No | 117,106 | 10,000 | 1.00 (ref.) | 1.08 (1.00-1.17) | 1.04 (0.95-1.13) | | 1.11 (1.02-1.20) | | 1.08 (0.98-1.20) | | 0.098 |  |
| Yes | 50,264 | 5,170 | 1.00 (ref.) | 0.94 (0.82-1.07) | 1.05 (0.92-1.20) | | 1.04 (0.91-1.19) | | 1.14 (0.98-1.32) | | 0.001 |  |
| Calcium and/or vitamin D combination |  |  |  |  |  | |  | |  | |  | 0.733 |
| No | 149,735 | 12,699 | 1.00 (ref.) | 1.06 (0.99-1.14) | 1.08 (1.00-1.16) | | 1.12 (1.04-1.20) | | 1.11 (1.02-1.22) | | 0.003 |  |
| Yes | 17,635 | 2,471 | 1.00 (ref.) | 0.83 (0.66-1.04) | 0.78 (0.63-0.98) | | 0.84 (0.67-1.04) | | 0.93 (0.74-1.18) | | 0.375 |  |
| Steroid |  |  |  |  |  | |  | |  | |  | 0.729 |
| No | 46,143 | 3,460 | 1.00 (ref.) | 1.10 (0.99-1.21) | 1.05 (0.94-1.17) | | 1.13 (1.00-1.27) | | 1.06 (0.85-1.31) | | 0.219 |  |
| Yes | 121,227 | 11,710 | 1.00 (ref.) | 0.99 (0.90-1.10) | 1.02 (0.92-1.12) | | 1.05 (0.96-1.16) | | 1.08 (0.97-1.20) | | 0.003 |  |
| Respiratory diseases |  |  |  |  |  | |  | |  | |  | 0.299 |
| No | 27,184 | 1,832 | 1.00 (ref.) | 1.13 (1.00-1.27) | 1.16 (1.00-1.34) | | 1.15 (0.98-1.36) | | 0.92 (0.64-1.33) | | 0.193 |  |
| Yes | 140,186 | 13,338 | 1.00 (ref.) | 0.97 (0.89-1.06) | 0.97 (0.89-1.06) | | 1.02 (0.93-1.11) | | 1.05 (0.95-1.16) | | 0.009 |  |
| Intra-abdominal infections |  |  |  |  |  | |  | |  | |  | 0.570 |
| No | 160,555 | 14,469 | 1.00 (ref.) | 1.04 (0.97-1.12) | 1.05 (0.98-1.13) | | 1.09 (1.02-1.17) | | 1.11 (1.02-1.21) | | 0.002 |  |
| Yes | 6,815 | 701 | 1.00 (ref.) | 1.00 (0.67-1.49) | 0.93 (0.62-1.39) | | 1.05 (0.71-1.55) | | 1.10 (0.72-1.69) | | 0.367 |  |
| Urinary tract infections |  |  |  |  |  | |  | |  | |  | 0.007 |
| No | 139,777 | 11,999 | 1.00 (ref.) | 1.05 (0.98-1.13) | 1.05 (0.98-1.14) | | 1.11 (1.03-1.20) | | 1.17 (1.07-1.28) | | <0.001 |  |
| Yes | 27,593 | 3,171 | 1.00 (ref.) | 0.79 (0.62-1.02) | 0.81 (0.63-1.04) | | 0.82 (0.64-1.04) | | 0.80 (0.62-1.03) | | 0.687 |  |
| Intestinal infectious diseases |  |  |  |  |  | |  | |  | |  | 0.339 |
| No | 135,091 | 12,090 | 1.00 (ref.) | 1.05 (0.98-1.13) | 1.06 (0.98-1.14) | | 1.09 (1.01-1.17) | | 1.11 (1.02-1.22) | | 0.008 |  |
| Yes | 32,279 | 3,080 | 1.00 (ref.) | 0.98 (0.77-1.25) | 0.97 (0.76-1.23) | | 1.06 (0.84-1.34) | | 1.08 (0.84-1.38) | | 0.059 |  |
| Skin, soft tissue, bone and joint infections |  |  |  |  |  | |  | |  | |  | 0.283 |
| No | 77,771 | 6,204 | 1.00 (ref.) | 1.02 (0.93-1.11) | 1.01 (0.92-1.11) | | 0.99 (0.90-1.09) | | 1.12 (0.99-1.27) | | 0.545 |  |
| Yes | 89,599 | 8,966 | 1.00 (ref.) | 1.05 (0.94-1.18) | 1.06 (0.94-1.18) | | 1.12 (1.00-1.25) | | 1.09 (0.97-1.24) | | 0.018 |  |
| Other infectious diseases |  |  |  |  |  | |  | |  | |  | 0.888 |
| No | 149,871 | 13,377 | 1.00 (ref.) | 1.05 (0.98-1.12) | 1.05 (0.98-1.13) | | 1.10 (1.02-1.18) | | 1.10 (1.01-1.20) | | 0.005 |  |
| Yes | 17,499 | 1,793 | 1.00 (ref.) | 0.79 (0.54-1.17) | 0.79 (0.54-1.16) | | 0.83 (0.57-1.20) | | 0.90 (0.62-1.32) | | 0.198 |  |

The aHRs were calculated by Cox proportional hazards regression after adjustments for multivariate variables. Model adjusted for age, gender, household income, Charlson comorbidity index, body mass index, systolic blood pressure, fasting serum glucose, total cholesterol, smoking status, alcohol intake, physical activity, diabetes, calcium and/or vitamin D combination, and steroid. The estimates were based on fully adjusted models.

Acronym: aHR, adjusted hazard ratio; CI, confidence interval; ref, reference.

**Supplementary Table S7. Stratified analysis of the association between the cumulative antibiotic days and the risk of osteoporotic fracture according to the prescription of calcium and/or vitamin D combinations among individuals who have been prescribed steroids.**

| Calcium and/or vitamin D combination | Cumulative days of antibiotics prescribed for 5 years before the index date | | | | | *P* for trend |
| --- | --- | --- | --- | --- | --- | --- |
|  | None | 1-14 days | 15-30 days | 31-90 days | ≥91days |  |
| No |  |  |  |  |  |  |
| Number of participants, n | 5,640 | 27,764 | 26,560 | 36,332 | 10,438 |  |
| Events, n | 421 | 2,222 | 2,370 | 3,570 | 1,039 |  |
| aHR (95% CI) | 1.00 (ref.) | 1.01 (0.91-1.12) | 1.05 (0.95-1.17) | 1.08 (0.98-1.20) | 1.08 (0.96-1.21) | 0.008 |
| Yes |  |  |  |  |  |  |
| Number of participants, n | 331 | 2,521 | 3,331 | 6,138 | 2,172 |  |
| Events, n | 52 | 362 | 437 | 876 | 361 |  |
| aHR (95% CI) | 1.00 (ref.) | 0.85 (0.64-1.14) | 0.77 (0.58-1.03) | 0.83 (0.62-1.09) | 0.95 (0.71-1.27) | 0.329 |

The aHRs were calculated by Cox proportional hazards regression after adjustments for multivariate variables. Model adjusted for age, gender, household income, Charlson comorbidity index, body mass index, systolic blood pressure, fasting serum glucose, total cholesterol, smoking status, alcohol intake, physical activity, and diabetes.

Acronym: aHR, adjusted hazard ratio; CI, confidence interval; ref, reference.

**Supplementary Table S8. Stratified analysis of the association between the cumulative antibiotic days and the risk of osteoporotic fracture according to the prescription of calcium and/or vitamin D combinations among individuals who have been diagnosed with diabetes.**

| Calcium and/or vitamin D combination | Cumulative days of antibiotics prescribed for 5 years before the index date | | | | | *P* for trend |
| --- | --- | --- | --- | --- | --- | --- |
|  | None | 1-14 days | 15-30 days | 31-90 days | ≥91days |  |
| No |  |  |  |  |  |  |
| Number of participants, n | 3,305 | 11,737 | 10,502 | 14,382 | 4,447 |  |
| Events, n | 248 | 954 | 1,038 | 1,528 | 485 |  |
| aHR (95% CI) | 1.00 (ref.) | 0.94 (0.82-1.09) | 1.05 (0.91-1.20) | 1.04 (0.90-1.19) | 1.06 (0.91-1.24) | 0.039 |
| Yes |  |  |  |  |  |  |
| Number of participants, n | 166 | 995 | 1,339 | 2,408 | 983 |  |
| Events, n | 22 | 128 | 200 | 376 | 191 |  |
| aHR (95% CI) | 1.00 (ref.) | 0.90 (0.57-1.41) | 1.04 (0.67-1.63) | 1.05 (0.68-1.62) | 1.37 (0.88-2.15) | 0.002 |

The aHRs were calculated by Cox proportional hazards regression after adjustments for multivariate variables. Model adjusted for age, gender, household income, Charlson comorbidity index, body mass index, systolic blood pressure, fasting serum glucose, total cholesterol, smoking status, alcohol intake, physical activity, and steroid.

Acronym: aHR, adjusted hazard ratio; CI, confidence interval; ref, reference.

**Supplementary Table S9. Association between the number of prescribed antibiotic classes and the risk of osteoporotic fracture among participants who were prescribed antibiotics.**

|  | Number of antibiotic classes prescribed during 5 years before the index date | | | | *P* for trend |
| --- | --- | --- | --- | --- | --- |
|  | 1 | 2 | 3 | ≥4 |  |
| Number of participants, n | 27,699 | 41,823 | 47,018 | 35,547 |  |
| Events, n | 2,205 | 3,790 | 4,428 | 3,704 |  |
| Person-years | 281,193 | 423,162 | 474,609 | 357,431 |  |
| aHR (95% CI) |  |  |  |  |  |
| Model 1 | 1.00 (ref.) | 1.06 (1.00-1.12) | 1.04 (0.99-1.10) | 1.11 (1.05-1.17) | 0.001 |
| Model 2 | 1.00 (ref.) | 1.05 (0.99-1.10) | 1.02 (0.97-1.08) | 1.08 (1.02-1.14) | 0.037 |
| Model 3 | 1.00 (ref.) | 1.04 (0.99-1.10) | 1.01 (0.96-1.06) | 1.06 (1.00-1.12) | 0.162 |

The aHRs were calculated by Cox proportional hazards regression after adjustments for multivariate variables. Model 1 adjusted for age, gender, household income, Charlson comorbidity index, body mass index, systolic blood pressure, fasting serum glucose, total cholesterol, smoking status, alcohol intake, and physical activity. Model 2 adjusted for diabetes, calcium and/or vitamin D combination, and steroid in addition to the variables in Model 1. Model 3 adjusted for infectious diseases (respiratory diseases, intra-abdominal infections, urinary tract infections, intestinal infectious diseases, skin, soft tissue, bone, and joint infections, and other infectious diseases) in addition to the variables in Model 2.

Acronym: n, number of people; aHR, adjusted hazard ratio; CI, confidence interval; ref, reference.

**Supplementary Table S10. Comparison of specific antibiotic class users and antibiotic non-users for osteoporotic fracture risk.**

| Exposure variable | Total | Events | Person-years | aHR (95% CI) |
| --- | --- | --- | --- | --- |
| Cephalosporins |  |  |  |  |
| Non-user | 15,283 | 1,043 | 156,238 | 1.00 (ref.) |
| Only-user | 10,814 | 837 | 110,047 | 1.02 (0.93-1.12) |
| Penicillins (including ampicillin and amoxicillin) |  |  |  |  |
| Non-user | 15,283 | 1,043 | 156,238 | 1.00 (ref.) |
| Only-user | 7,759 | 580 | 79,291 | 1.00 (0.90-1.10) |
| Fluoroquinolones |  |  |  |  |
| Non-user | 15,283 | 1,043 | 156,238 | 1.00 (ref.) |
| Only-user | 7,087 | 624 | 71,211 | 1.01 (0.91-1.12) |
| Macrolides |  |  |  |  |
| Non-user | 15,283 | 1,043 | 156,238 | 1.00 (ref.) |
| Only-user | 1,572 | 125 | 15,930 | 1.00 (0.82-1.20) |
| Tetracyclines |  |  |  |  |
| Non-user | 15,283 | 1,043 | 156,238 | 1.00 (ref.) |
| Only-user | 425 | 34 | 4,320 | 1.02 (0.72-1.43) |
| Lincosamides |  |  |  |  |
| Non-user | 15,283 | 1,043 | 156,238 | 1.00 (ref.) |
| Only-user | 42 | 5 | 396 | 1.60 (0.66-3.86) |

The aHRs were calculated by Cox proportional hazards regression after adjustments for multivariate variables. Model adjusted for age, gender, household income, Charlson comorbidity index, body mass index, systolic blood pressure, fasting serum glucose, total cholesterol, smoking status, alcohol intake, physical activity, diabetes, calcium and/or vitamin D combination, and steroid.

Acronym: aHR, adjusted hazard ratio; CI, confidence interval; ref, reference.
